# Nanostructured gold in ancient Ayurvedic calcined drug ‘*swarnabhasma*’

**DOI:** 10.1016/j.jaim.2021.06.017

**Published:** 2021-10-22

**Authors:** Trupti Patil-Bhole, Asmita Wele, Ramacharya Gudi, Kapil Thakur, Shailesh Nadkarni, Rajendra Panmand, Bharat Kale

**Affiliations:** a Department of Rasashastra and Bhaishajyakalpana, College of Ayurved, Bharati Vidyapeeth (Deemed to be University), Pune 411043, India; b Shree Dhootapapeshwar Limited, Panvel, Mumbai, Maharashtra, India; c Centre for Materials for Electronics Technology, Thrissur, 680581, Kerala, Under Ministry of Electronics and Information Technology, India; d Centre for Materials for Electronics Technology, Pune 411008, Maharashtra, Under Ministry of Electronics and Information Technology, India

**Keywords:** *Swarnabhasma*, Gold, *Bhasma*, *Rasashastra*, *Ayurveda*, Characterization, Cancer, *Parad*, Catalytic, Mercury, Nanoparticles

## Abstract

**Background:**

*Swarnabhasma* (calcined gold) is a famous ancient Ayurvedic medicine. However, its detail characteristic investigations are very limited.

**Objective:**

Herein, investigation of *swarnabhasma* is demonstrated using ancient and ultramodern techniques to understand the physicochemical nature of this drug, and to understand whether the mercury [*Parada*] used during preparation method marks its presence in *swarnabhasma*.

**Materials and methods:**

The investigated *swarnabhasma* was prepared by repeated incinerations of Au–Hg-Lemon juice amalgamation and sulphur. The *bhasma* was tested by all traditional tests of *rasashastra*. It was characterized by X-ray diffraction (XRD), Field Emission Scanning Electron Microscope (FE-SEM), Field Emission Transmission Electron Microscopy (FE-TEM), Inductively Coupled Plasma Atomic Emission Spectroscopy (ICP-AES), Energy Dispersive X-ray Fluorescence (EDXRF), Fourier Transform Infrared Spectroscopy (FTIR), and gravimetric analysis.

**Results:**

Traditional tests of *rasashastra* were complied by the sample. XRD confirms that *swarnabhasma* consists of principally pure gold at nanoscale. FE-SEM showed agglomerated particles. FE-TEM showed that *swarnabhasma* contains highly crystalline nanostructured gold comprised with spherical gold nanoparticles of size, 5–20 nm. ICP-AES exhibited absolute absence of Hg and presence of Au, Si, Ag, Al, Ca, Cu, Fe, K, Mg, Mn, Na, P, Sr, Ti, and Zn. EDXRF confirmed the absence of mercury and confirmed the presence of Au, Si, Zr, Nb, S, Cl, K, Ca, Fe, and Ni. FTIR confirmed presence of water molecules adsorbed over surface of *bhasma*. Gravimetric analysis confirmed presence of 95% gold.

**Conclusion:**

Nano-structuring of gold enhances the surface area as well as activity. The present investigation shows that the entire process from *rasashastra* confers the unique nanostructure to gold and same is responsible for its medicinal potential. This nanomedicine is highly stable, which is specified as *niruttha* and *apunarbhava* in *rasashastra*.

## 1 Introduction

The noble metal gold (Au), has been known from time immemorial for its medicinal uses. Ancient manuscripts of *rasashastra* recommend calcined gold (*swarnabhasma*) as a drug of rejuvenation and longevity [1,2].

*Bhasmas* prepared by traditional Ayurvedic processes have been regarded as a most ancient form of nanomedicine [[3], [4], [5], [6]]. *Swarnabhasma* is an ancient Ayurvedic medicine, a stable form of gold characterized by lightness. In recent years, nanoparticles are manufactured by chemical reduction of Au (III) ions using various reducing agents like citric acid [7]. The challenges of toxicity issues have been evident with synthetically made gold nanoparticles (AuNPs) [8].

In various Ayurvedic manuscripts, around twenty-five different preparation methods of *bhasma* from gold are documented. These processes involve use of substances like mercury, sulfur, lead, cinnabar, mercury sulphide, asaphoetida, lemon juice, etc, apart from gold, and calcined repeatedly. Previous studies have reported characterization of *swarnabhasma* prepared by different methods. Varied amounts of gold percentage were noted in different samples prepared by different methods [[9], [10], [11], [12], [13], [14]]. Varied amount of gold was evident in previously investigated *swarnabhasma* samples prepared by different methods. Elemental percentage of gold in *bhasma* was 20.34% [9], ∼90% [(ii), [10], (i)], and ∼52% [13]. Variation in ingredients and processing has led to variable elemental composition of different samples of *swarnabhasma*. For instance, some of the processing ingredients add up to the final *bhasma*, and some get evaporated. Interestingly, maximum gold percentage was evident in *swarnabhasma* prepared using mercury (*parada*) and gold [10,14]. There is inadequate evidence to understand the nature of particles of *swarnabhasma*. In the present work, the method of *swarnabhasma* preparation involved use of gold-mercury amalgamation, juice of *Citrus medica* and sulfur.

The liquid metal, mercury (*parada*), is valued as an important ingredient in medicine and alchemy, which has contributed to the history of science since ages [15,16]. Mercury has been used extensively during conversion of metals into *bhasma*. It is one of the first metals known to mankind, and one of important metals of alchemy and *‘rasashastra’*, the technique of metallic medicine [17,18]. This paper reports about the gold *bhasma* manufactured by use of mercury. Whether the mercury marks its presence in the final formulation, was an important research question during the present study of characterization. To our knowledge the unique preparation method of *swarnabhasma* involving mercury has not been explored completely which in turn is responsible for the structure of the final product. There have been some controversies regarding the use of mercury in Ayurveda, and presence of mercury in medicines [[19], [20], [21]]. There are systematic processes and guidelines of *rasashastra* through which mercury should be used to formulate medicine. *Swarnabhasma* is a *rasashastra-*based medicine, and with this study, an attempt to highlight the importance of mercury in its preparation was made.

We carried out an extensive study of *swarnabhasma* with Field Emission Transmission Electron Microscopy (FE-TEM) and found that the presented method yields formation of unique nano-gold assemblies which have been safely used as a medicine since ancient times.

We carried out and demonstrated the characterization by X-ray diffraction (XRD), Field Emission Scanning Electron Microscope (FE-SEM), FE-TEM, Inductively Coupled Plasma Atomic Emission Spectroscopy (ICP-AES), Energy Dispersive X-ray Fluorescence (EDXRF), Fourier Transform Infrared Spectroscopy (FTIR), and gravimetric test. The investigations were aimed to assess the total gold percentage, elemental composition, crystal structure, morphology, particle size, and nature of particles. Ayurvedic confirmatory tests were carried out including ‘floating test’ of particles (*varitaratva*) which confirms lightness, regarded as the most crucial test for metallic *bhasma*. This test ensures safe use of *bhasma* in human body. Other traditional tests viz, *Niswadu, Nishchandra, Unam, Rekhapurnatva, Nirdhuma,* *Niruttha,* and *Apunarbhavatva* were carried out. The traditional tests of *swarnabhasma* are simple and reliable techniques to ensure that the process of *bhasma* formation is complete, which confirms suitability for further medicinal use in 15 mg–240 mg dose range.

## 2 Materials and methods

### 2.1 Preparation of *swarnabhasma* (calcined gold)

*Swarnabhasma* (gold *bhasma*) was manufactured by the industry ‘Shree Dhootapapeshwar Limited’, in Maharashtra, India [22]. The detailed process of manufacturing is reported here. Pure 24 carat gold foil, mercury, and sulfur were procured from authentic sources in Mumbai. The three substances were processed by specific ‘purification’ (*shodhan*) techniques reported literature of *rasashastra* [23,24]. Purified denotes ‘processed by specific methods of *rasashastra’*. Following processes were carried out for purification of gold, mercury, and sulfur.

Pure gold foil was cut into small grain-sized pieces. Gold pieces were heated until red hot and quenched in decoction of *Bauhinia variegata* L (Leguminosae), thrice, followed by air drying. For purification, mercury (99.9% pure) was triturated with a paste of garlic cloves (*Allium sativum* L., Amaryllidaceae) and powdered rock salt for 7 days, and further washed with lemon juice and water [25]. For purification, sulfur was melted with cow ghee (clarified butter) and poured into cow milk seven times [26]. It was then washed with warm water, dried, and powdered using a grinder.

Materials required for manufacture of *swarnabhasma* included purified gold (1 kg), purified mercury (2 kg), purified sulfur (16 kg, in divided quantity per *puta*) and juice of *C.* *medica* (QS). An iron mortar and pestle was used for pounding process. Earthen crucibles and Fuller’s earth were used for preparing encapsulated case (*sharav samputa)* of material to be calcined. Cow dung cakes were used as fuel during the process.

#### 2.1.1 Process of incineration

**Fig. 1:**
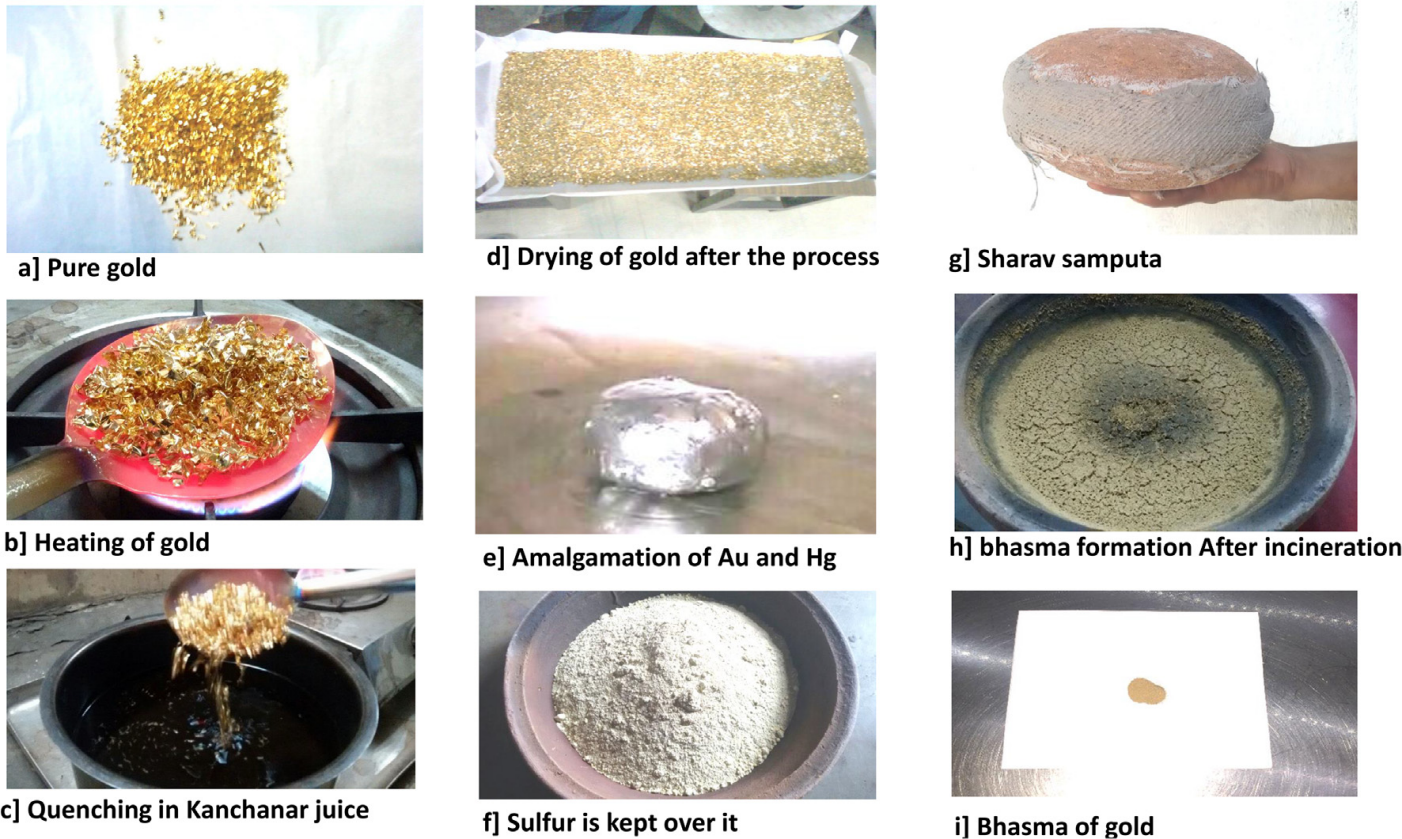
Figure showing making of *swarnabhasma* by traditional process of *rasashastra*. a) Pure gold, b) Heating of gold, c) Quenching them in *kanchanar* decoction d) Drying of gold, e) Amalgam of Au and Hg, f) Au–Hg amalgam and sulfur is placed in earthen saucer, g) *Sharav samputa*, h) *Bhasma* formation after repeated incineration, i) Sample of *swarnabhasma*.

Fig. 1 demonstrates the process of manufacturing of *swarnabhasma*. Detoxified gold and mercury (1:2) and juice of *C.* *medica* (QS) were pounded to form an amalgam in a slightly warm mortar pestle of cast iron. Amalgam was placed in an earthen crucible with equal amount of sulfur powder, and covered from above by another earthen crucible and the joint was sealed by an aqueous paste of Fuller’s earth and cotton cloth. The seal was allowed to dry, leaving a compact space available for reaction inside the crucibles. This case was heated with 30 cow-dung cakes in a pit, called *puta*, reaching gradually up to a temperature of 900 °C. The assembly was allowed to self cool. Contents were collected by breaking the seal carefully and were triturated to homogeneity. Further, the content was calcined again in the presence of equal amount of sulfur, in a similar way for 14 times. The mixture was converted into a powdered form ‘*swarnabhasma’* (Fig. 1, Fig. 9D).

**Fig. 2:**
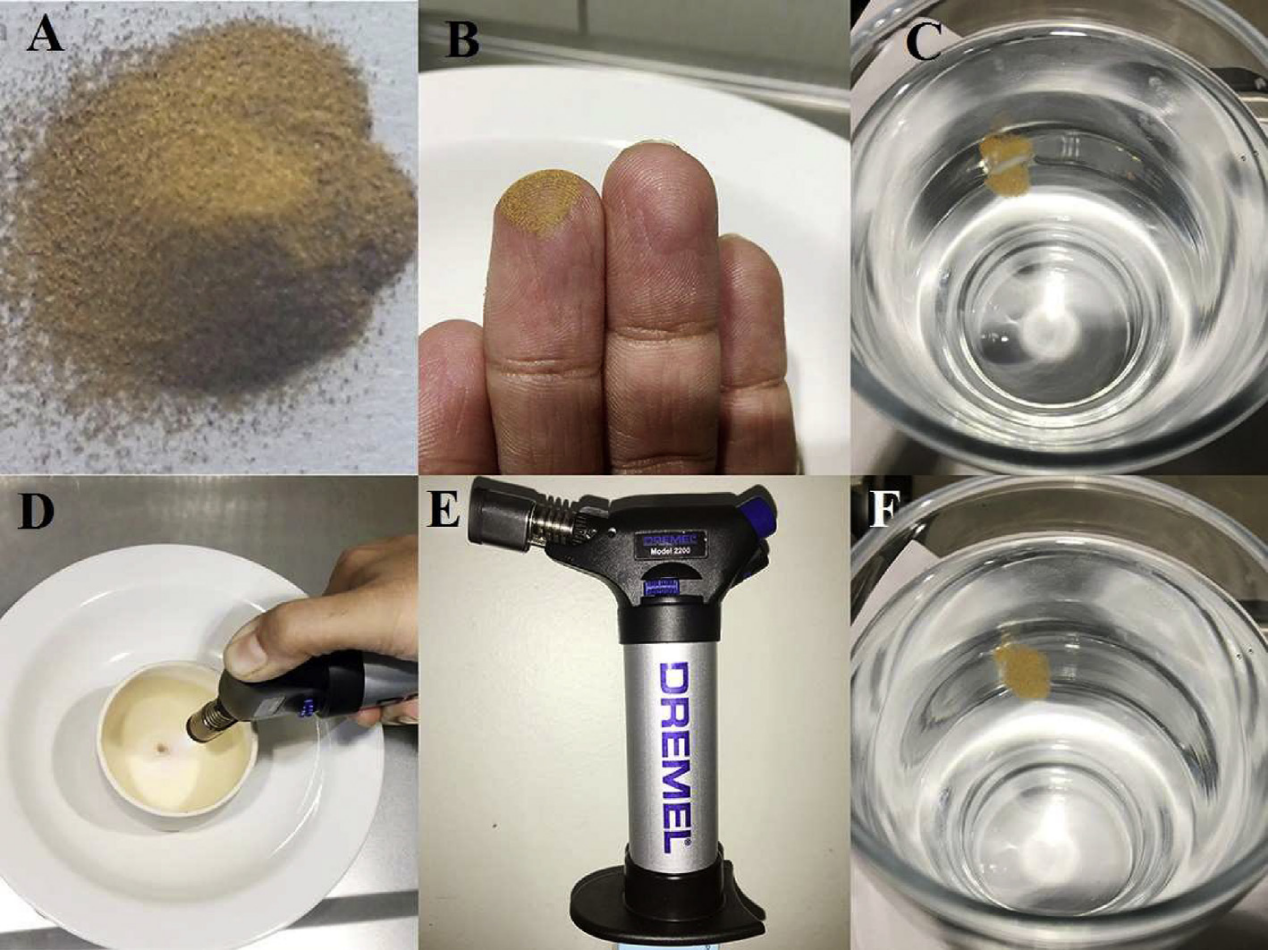
Shows images of A) *Swarnabhasma* (gold *bhasma*), B) Fine particles of the *bhasma* getting lodged in fine finger lines (*rekhapurnatva)*, C) Rice grain floating over the *bhasma* layer over water (*unama)*. D) No fume production on heating by strong butane flame (*nirdhuma*), E) Dremel versaflame butane torch used for heating during *nirdhuma, niruttha* and *apunarbhava* tests and F) *Bhasma* particles floating over water surface (*varitaratva*).

### 2.2 Ayurvedic tests

**Fig. 3:**
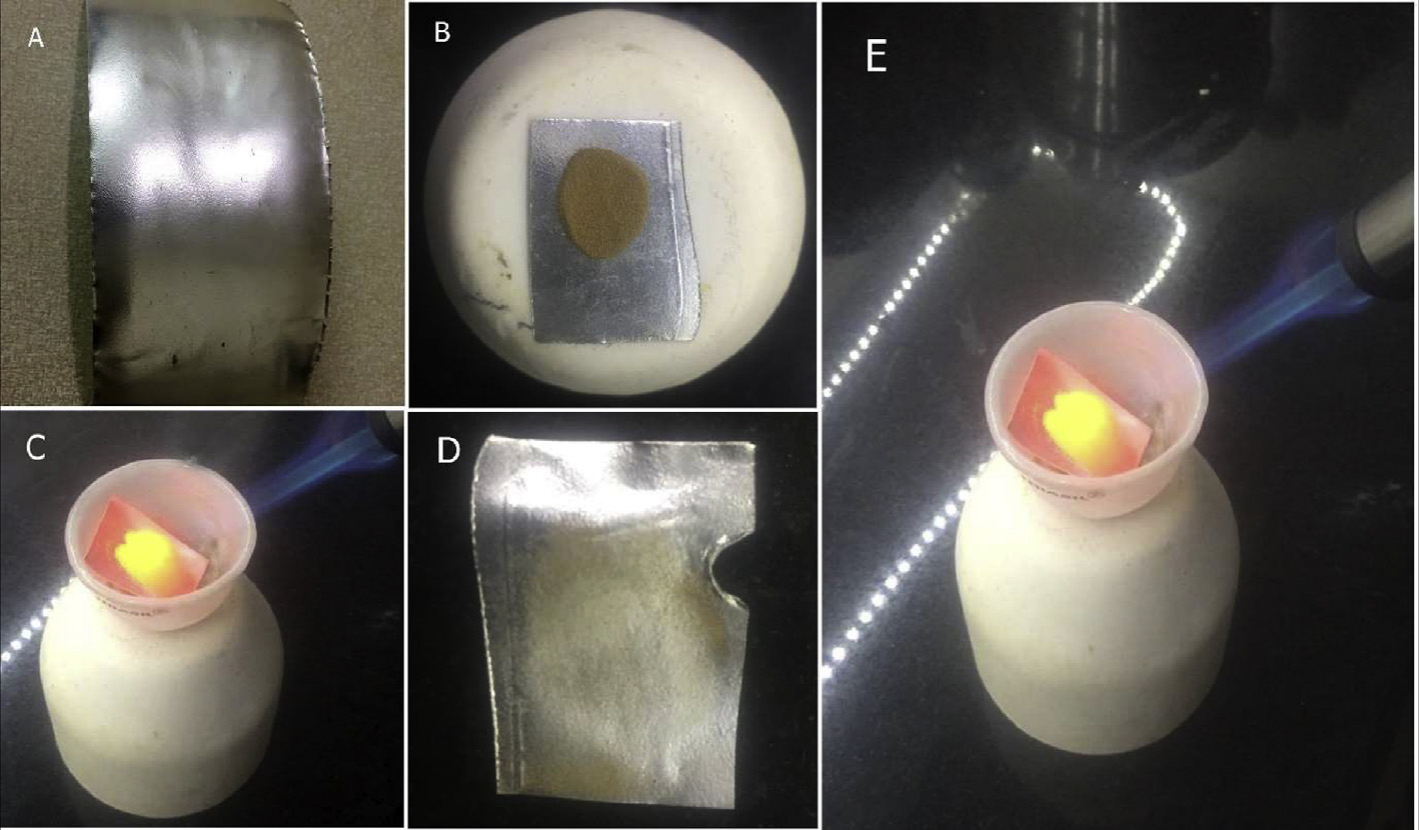
*Niruttha* test*:* A) Pure silver sheet, B) *Swarnabhasma* kept over silver sheet, C) Heating by flame in a crucible, D) Finally no weight change in silver, no adhesions over it after heating. E) This is enlarged view during *niruttha* test showing silver sheet and *swarnabhasma* being heated by Dremel versaflame torch, after which there was no change in appearance of *bhasma*.

**Fig. 4:**
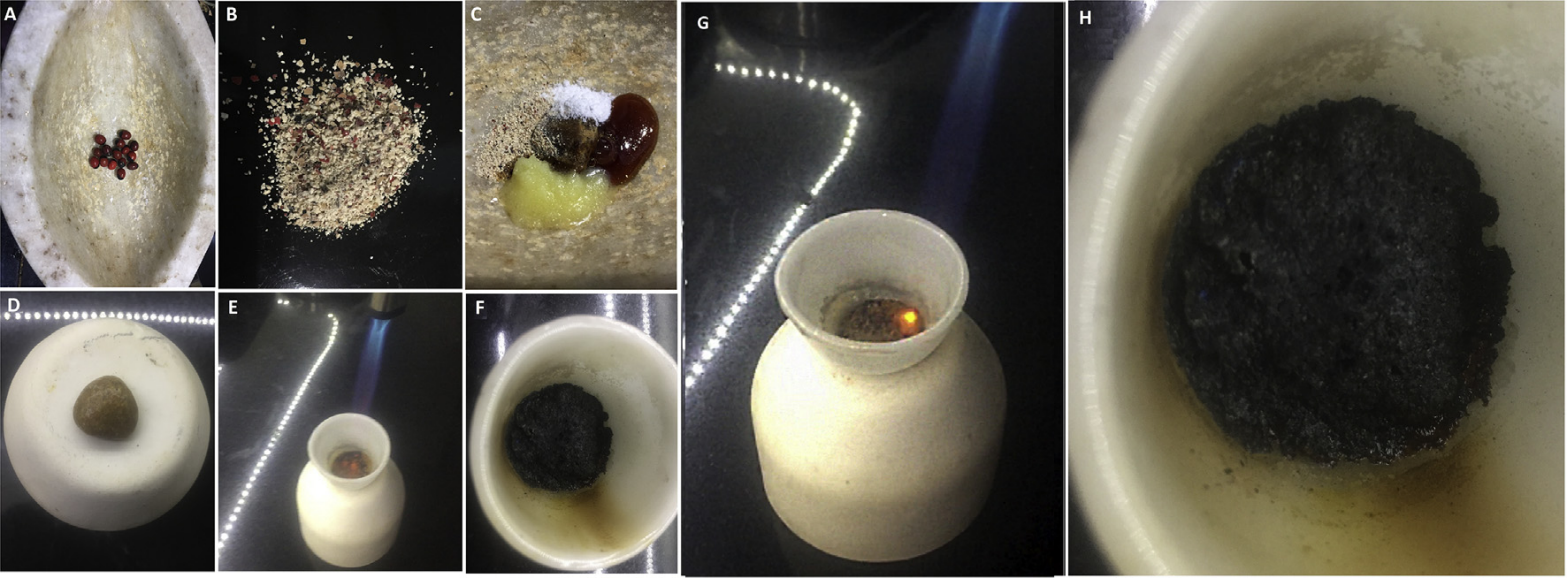
*Apunarbhavatva* test: A) Seeds of *Abrus precatorus* (*gunja*), B) Powder of the seeds, C) Mixing of *Abrus precatorus* seed powder, *ghrita*, honey (*madhu)*, borax (*tankana*) and jaggery (*guda*), D) Formation of bolus of *swarnabhasma* and all these ingredients, E) Heating of bolus in a crucible, F) Charred mixture showing no shiny particles, and no relapse of shiny gold metal., G) This is enlarged view of the bolus being charred, H) This is an enlarged view of 4F showing black colored charred mixture, no any shiny gold particles can be observed.

Traditional tests of *rasashastra* were carried out to confirm *bhasma* formation [Fig. 2, Fig. 3, Fig. 4]. *Bhasma* particles were tested by checking its colour, odor, and taste (*niswadu* test). The *bhasma* was observed in sunlight for any presence of shiny particles (*nishchandra* test). The *bhasma* particles were tested for lightness, confirmed by their characteristic ability to float over water. This was tested by sprinkling *bhasma* over the surface of still, distilled water in a glass beaker (*varitaratwa*) [27, 8/27]. A rice grain was placed over the layer of *bhasma* to check if it floats over (*unam*) [27, 8/30]. The sample was heated by a strong butane flame of 1200 °C produced by Dremel Versaflame for 10 min (*nirdhuma*). *Niruttha* and *apunarbhavatva* tests were carried out as follows.

*Apunarbhava* – *Swarnabhasma* (200 mg) was mixed with a paste (5 gm) of *ghrita* (cow’s ghee), *madhu* (honey), *guda* (jaggery), *gunja* (seeds of *Abrus precatorus*), *tankana* (borax) and the bolus was incinerated using a butane flame. The process did not yield shiny metallic particles in the burnt mass. Hence, the *bhasma* was confirmed and passed the test of ‘*apunarbhava’* [27, 8/29].

*Niruttha* test - In this test, pure silver [99.9%] and gold *bhasma* were placed in a crucible and subjected to heat by a butane flame. The temperature should reach till that of *puta* given during *bhasma* making i.e., 900 °C. After the test, silver was checked for any adhesions and weight gain. If there was gain in weight of silver or there were adhesions over silver, then *bhasma* was considered as *utthapita*. If there was no change in the weight of silver, the *bhasma* being tested was considered as *Niruttha* [27, 8/31].

*Rekhapurnatva* was assessed by checking whether the fine particles get stuck in fine lines of fingers [27, 8/28].

### 2.3 Advanced physicochemical tests

#### 2.3.1 XRD

A sample of *swarnabhasma* was examined by XRD (Bruker AXS, D 8 advance series, Germany). The sample of *bhasma* was spread onto a double-side tape with a spatula, and then placed on a PMMA sample holder. All peaks were recorded on the computer.

#### 2.3.2 FE-SEM

FE-SEM instrument of Alert, Hitachi, S4800 (Japan) was utilized for observation of morphology of *bhasma*. The sample was prepared by mounting a drop of *swarnabhasma* mixed in ethanol on carbon-coated copper grid and allowing the drop to dry in air.

#### 2.3.3 FE-TEM

FE-TEM of JEM- JEOL 2200 FS, USA, was used to get geometrics of the particles of *swarnabhasma*. The *bhasma* was mixed with ethanol for a satisfactory dispersal for imaging by FE-TEM. It was a challenge to prepare the sample due to the characteristic floating tendency and aggregate formation behavior of gold *bhasma* particles.

#### 2.3.4 ICP-AES

ICP-AES of Spectro-Analytical instruments, GmbH, Germany was utilized to check elemental composition. All chemicals of ultrapure grade were used.

#### 2.3.5 EDXRF

EDXRF of make Thermo Fishers; Model – ARL Quant’ X, was used, for qualitative analysis of elements present in *swarnabhasma*.

#### 2.3.6 FTIR

FTIR instrument model Spectrum 10 of PerkinElmer, was utilised for this study. The FTIR spectrometer collected high spectral resolution data for *swarnabhasma* over a wide spectral range.

#### 2.3.7 Gravimetric method for estimation of gold [28]

Quantitative estimation of gold was done by gravimetric method from Vogel’s quantitative chemical analysis. Five grams hydroquinone was dissolved in a 100 ml volumetric flask. Hundred milligrams of *swarnabhasma* was placed in a 250 ml beaker. Ten millilitre aqua regia was added to it and digested on a hot plate till the solution was free from nitric acid. Thirty millilitre of nitric acid was added and the compound was boiled for a few minutes. Excess of 5% quinol solution (3 ml for every 25 mg of gold) was added and the solution was boiled for 20 min. The solution was allowed to cool and filtered through a Whatman filter paper no 42. The filter paper was washed with hot water till all the fine particles of gold transferred into the ash-less filter paper. The paper was transferred in a silica crucible, and it was burnt off (ignited) to constant weight [28]. The percentage of gold was calculated in percentage.

## 3 Results

### 3.1 Ayurvedic tests (Traditional tests of *rasashastra*)

The investigated *swarnabhasma* was light brown in color, having no odour and no taste (*nisvadu)*. When the *bhasma* was exposed to sunlight, it was devoid of shining particles hence, confirming ‘*nishchandra’* character.

*Swarnabhasma* was *varitara* i.e., floating test was successful as *bhasma* particles floated over water when sprinkled over the surface of still, distilled water in a glass beaker (*varitaratva*). None of the particles sank. A rice grain placed over the layer of *bhasma*, stayed floating without sinking (*unam*). The particles of *bhasma* got lodged in the fine lines of the finger when the *bhasma* was rubbed between the fingers (*rekhapurnatva*). The sample showed no fumes and no change in colour or appearance of the *bhasma* on being heated by a strong butane flame (*nirdhuma*) was observed. There were no adhesions to silver and no weight change in silver when gold *bhasma* was heated with silver in *niruttha* test [Fig. 3]. The silver sheet melted from one side, but no weight change was observed after the test. There was no relapse of gold metal in *apunarbhava* test, as the charred bolus of *bhasma* with powder of *A. precatorus* seeds, *ghrita*, honey, borax, and jaggery did not show any shiny particles when observed in sunlight [Fig. 4].

All Ayurvedic confirmatory tests successfully indicated confirmation of thorough conversion of gold metal into medicinal gold *bhasma*.

### 3.2 Advanced physicochemical tests

#### 3.2.1 XRD

**Fig. 5:**
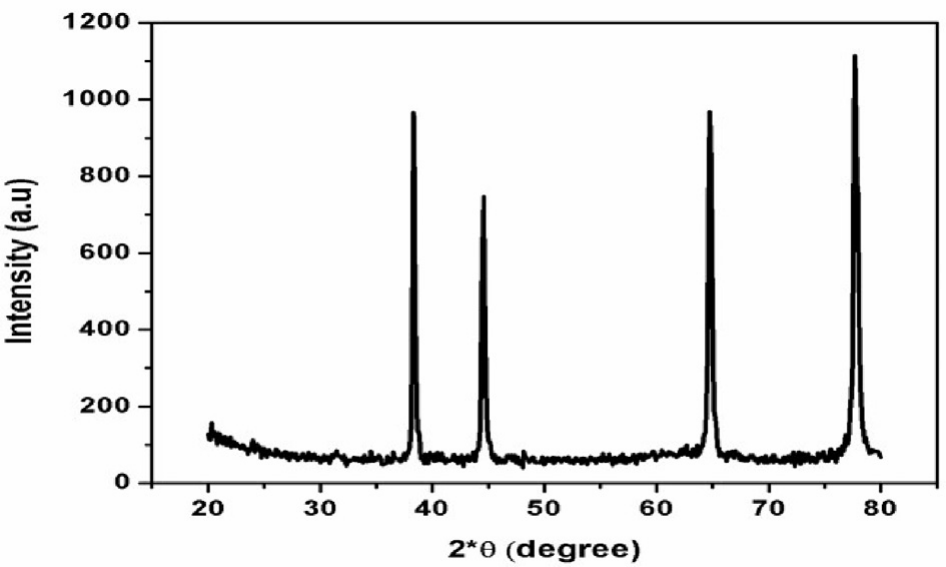
X-ray Diffraction pattern (XRD) of *swarnabhasma*.

The XRD pattern (Fig. 5) of investigated *swarnabhasma* showed four intense peaks of pure gold in the whole spectrum of 2θ values ranging from 20 to 80°. The presence of intense peaks of nanoparticles (1 1 1), (2 0 0), (2 2 0) and (3 1 1) appeared which are indexed as crystalline gold face centered cubic phase. The standard XRD pattern of *swarnabhasma* was similar to JCPDS (Joint Committee on Powder Diffraction Standards) data no: 01–1174 and also similar to previous study of gold *bhasma* [14]. Peak (3 1 1) shows the faceted growth of gold nanoparticles. XRD confirms that *swarnabhasma* comprises of pure gold.

**Fig. 6:**
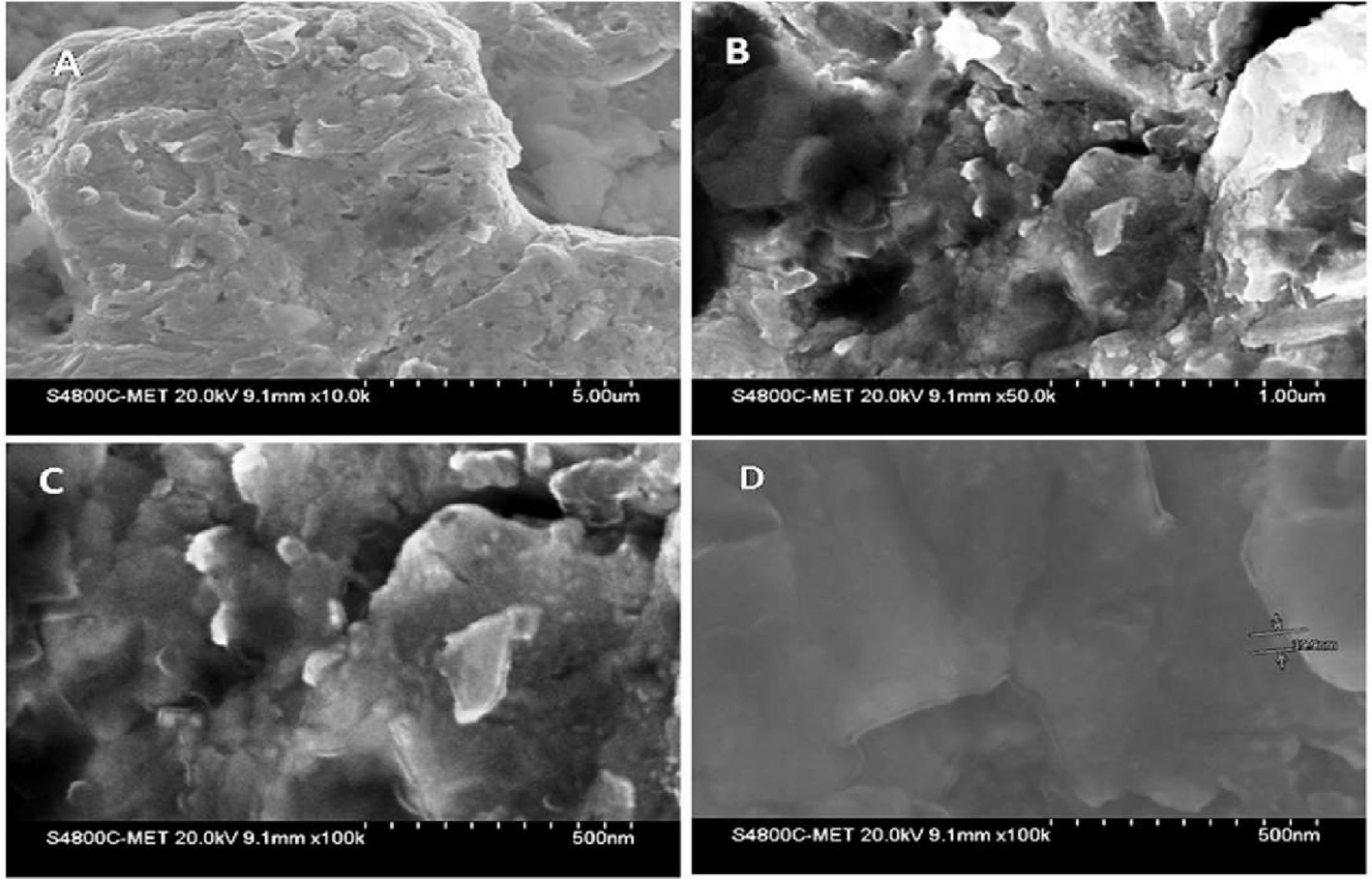
This shows the FE-SEM micrographs of *swarnabhasma*. Fig. 6A and B depicts the morphology of *swarnabhasma*. Fig. 6C and D represent the magnified images of the sample which depict the cluster of nanoparticles.

#### 3.2.2 FE-SEM

The morphology of the material was analyzed using FE-SEM. Fig. 6 shows the FE-SEM micrographs of synthesized *swarnabhasma*. Fig. 6A–B depicts the agglomerated morphology of aqueous mediated gold *bhasma*. The size is very small; hence, we investigated further by FE-TEM.

#### 3.2.3 FE-TEM

**Fig. 7:**
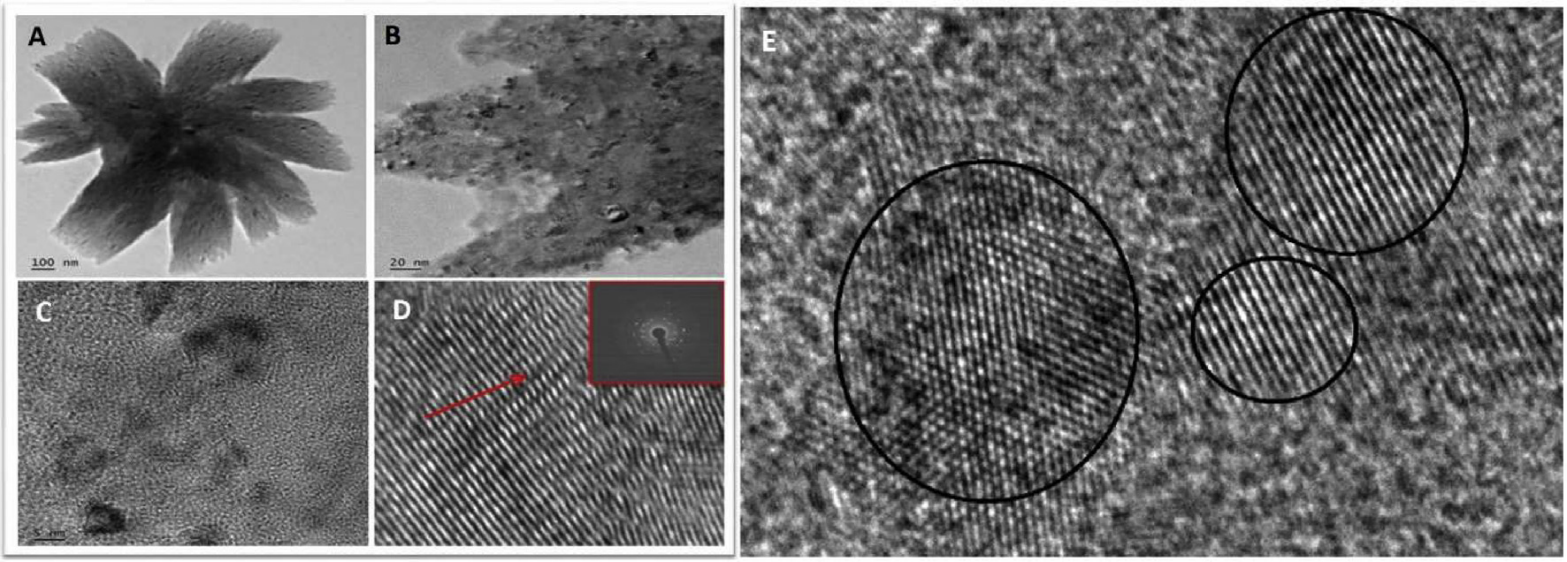
FE-TEM images of *swarnabhasma*. Fig. 7A and B are low magnification FE-TEM images of *swarnabhasma* which clearly show flower-like morphology of size 500–600 nm. Fig. 7C shows high magnification TEM images of *swarnabhasma* which clearly shows spherical particle of gold with size 5–20 nm. Fig. 7D is cropped image of single gold nanoparticle in investigated *swarnabhasma*. From Fig. 7D, it is clear that the growth of nanoparticles is along the (111) plane. Electron diffraction pattern is shown in inset of Fig. 7D which confirms the crystalline structure of the *bhasma*. Fig. 7G shows the gold nanoparticles of polycrystalline nature.

FE-TEM confirmed the presence of gold nanoparticles of 5–20 nm size in *swarnabhasma* [Fig. 7].

#### 3.2.4 ICP-AES

From the ICP-AES, it is evident that mercury and sulfur are not present in investigated *swarnabhasma*. We specifically checked these two elements as they were a part of the manufacturing process. ICP-AES showed presence of Au, Ag, Al, Ca, Cu, Fe, K, Mg, Mn, Na, P, Si, Sr, Ti, and Zn.

#### 3.2.5 EDXRF

**Fig. 8:**
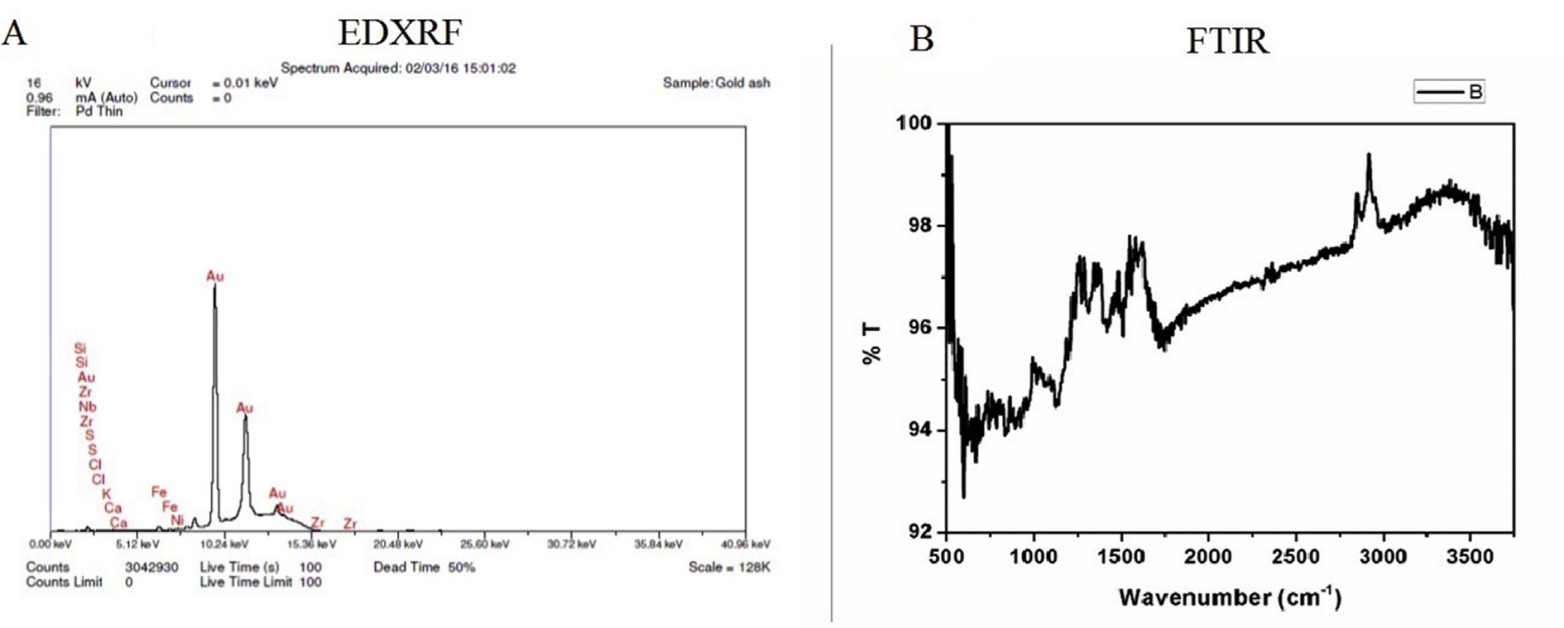
Fig. 8A is the EDXRF results and 8B exhibits FTIR pattern of investigated *swarnabhasma*.

EDXRF [Fig. 8 A] confirmed the absence of mercury. It confirmed the presence of Au, Si, Zr, Nb, S, Cl, K, Ca, Fe, Ni. The peaks of gold are prime, and other elements are in trace.

#### 3.2.6 FTIR

FTIR [Fig. 8 B] confirmed presence of water molecules over the sample. No other functional group could be ascertained.

#### 3.2.7 Gravimetric test

From investigated sample of *swarnabhasma,* 95% gold was obtained by gravimetric method [27].

## 4 Discussion

The investigated sample of *swarnabhasma* was prepared by incinerations of Au–Hg amalgam prepared in the presence of citrus juice and further addition of sulfur. Mercury is believed to play an important catalytic role during *bhasma-*making process. Gold being a noble metal resists any oxidation and hence, it’s a challenge to convert it to *bhasma,* a powder form. Interestingly, absence of mercury in the final product, *swarnabhasma,* was evident by ICP-AES and EDXRF.

EDXRF is an X-ray fluorescence technique used for elemental analysis. It is a non-destructive analytical technique. An X-ray beam with enough energy to affect the electrons in the inner shells of the atoms in a sample is created by an X-ray tube inside the handheld analyser. Light elements (below Al) have very limited sensitivity for detection by this technique. This is a surface-based technique unlike the destructive techniques like ICP-MS and ICP-AES for elemental analysis. This is because it is a surface sensitive method. The intension to carry out EDXRF and ICP-AES was detection of elements in *swarnabhasma* and especially to check for the presence or absence of mercury (*Parada*) which has been used in the manufacturing process of this sample.

FTIR identifies chemical bonds in a molecule by producing an infrared absorption spectrum. The spectra produce a profile of the sample, a distinctive molecular fingerprint that can be used to screen and scan samples for many different functional groups. FTIR is an effective analytical instrument for detecting functional groups and characterizing covalent bonding information. The presence of organic functional groups is important. Only water molecules could be identified in the investigated sample. Water molecules may not be a part of *swarnabhasma*, but adsorbed over it after its fabrication. FTIR showed water molecules or OH groups that are adsorbed on the surface after its fabrication. This technique is used for detecting any attached functional groups, for instance in case of AuNPs prepared by non–Ayurvedic contemporary methods involving green synthesis. In case of *swarnabhasma,* no other functional group could be identified, because the calcination process (*puta*) involved high temperatures.

During the process of incineration, from the pounded gold-mercury amalgamate the mercury might be escaping in vapor form and sulfur might be escaping in sulfur dioxide form. There is most likely, a layer-by-layer conversion of gold into *bhasma* particles during the 14 incinerations. Sulfur was added each time in the closed casing comprising of previously calcined and further triturated gold compound. *Swarnabhasma* prepared in this way is stable and does not revert to its original metallic state. The mercury amalgam may be acting as a growth directing agent. It is hypothesized that mercury may get vaporized and stick itself to the pores of earthen vessel, which needs to be confirmed by analysis of earthen vessels. Further research is needed for checking this. The techniques of mercury determination can be used for such analysis.

Powder XRD method is the best known sensitive method as a phase characterization tool. XRD confirms *swarnabhasma* to be pure gold, and not its oxide or sulphide. Faceted growth of gold nanoparticles is evident by XRD.

The nanosize and agglomeration of particles is evident by TEM and FE-SEM. The ability of nanoparticles to form larger agglomerates or any assembly has been extensively noted in literature [29]. The agglomerates are aggregation of gold nanoparticles in large micrometric size. This aggregation is due to sinterization by high temperatures and the particles are bound by certain forces. The glomeration tendency of particles of gold *bhasma* is significant and may have a role in its action, and our use of *anupanas*. It is evident from a previous research study that when administered intravenously into a mouse model, gold nanoparticle superstructures of reversible agglomerates and irreversible aggregates demonstrate significant differences in organ and cellular distribution compared with the primary particle building blocks [30]. The main advantage of *swarnabhasma* is its stability and its claimed safety as understood by use since ancient times as a medicinal agent. Stability is known by the irreversible nature of particles. Even after a long-term storage, it is known to be irreversible. The two tests *niruttha* and *apunarbhavatva* indicate the irreversible nature of gold *bhasma*. *Apunarbhava* test is a unique test which depicted irreversible nature of *bhasma* in this study. These are specific iatrochemistry based tests used to check if the *bhasma* (conversion from raw to consumable) is complete. If the *bhasma* is not properly formed, there is a relapse of metal particles in *apunarbhavatva* test. The test makes use of charring the *bhasma* with bolus of *A. precatorus* seed powder (*gunja*), cow’s ghee, honey (*madhu*), borax (t*ankana*) and jaggery (*guda*). This is nothing but extracting metal from its compound by traditional iatro-chemical way which is an unexplored mechanism.

Drug delivery of *swarnabhasma* is done in a specific way in which *bhasma* particles are mixed with media like honey, ghee, etc. The hierarchical nano-structured gold *bhasma* particles have high surface area and when mixed with honey or ghee, presumably release their agglomeration and get uniform dispersal and separation of particles, resulting in larger surface area. Further research on these drug delivery systems of Ayurveda needs to be done. Around 60 accompanying media or medicaments as *anupana* or yoga of *swarnabhasma* are available in ancient Ayurvedic literature. This includes honey, ghee, medicated milk, medicated clarified butter, and many more.

**Fig. 9:**
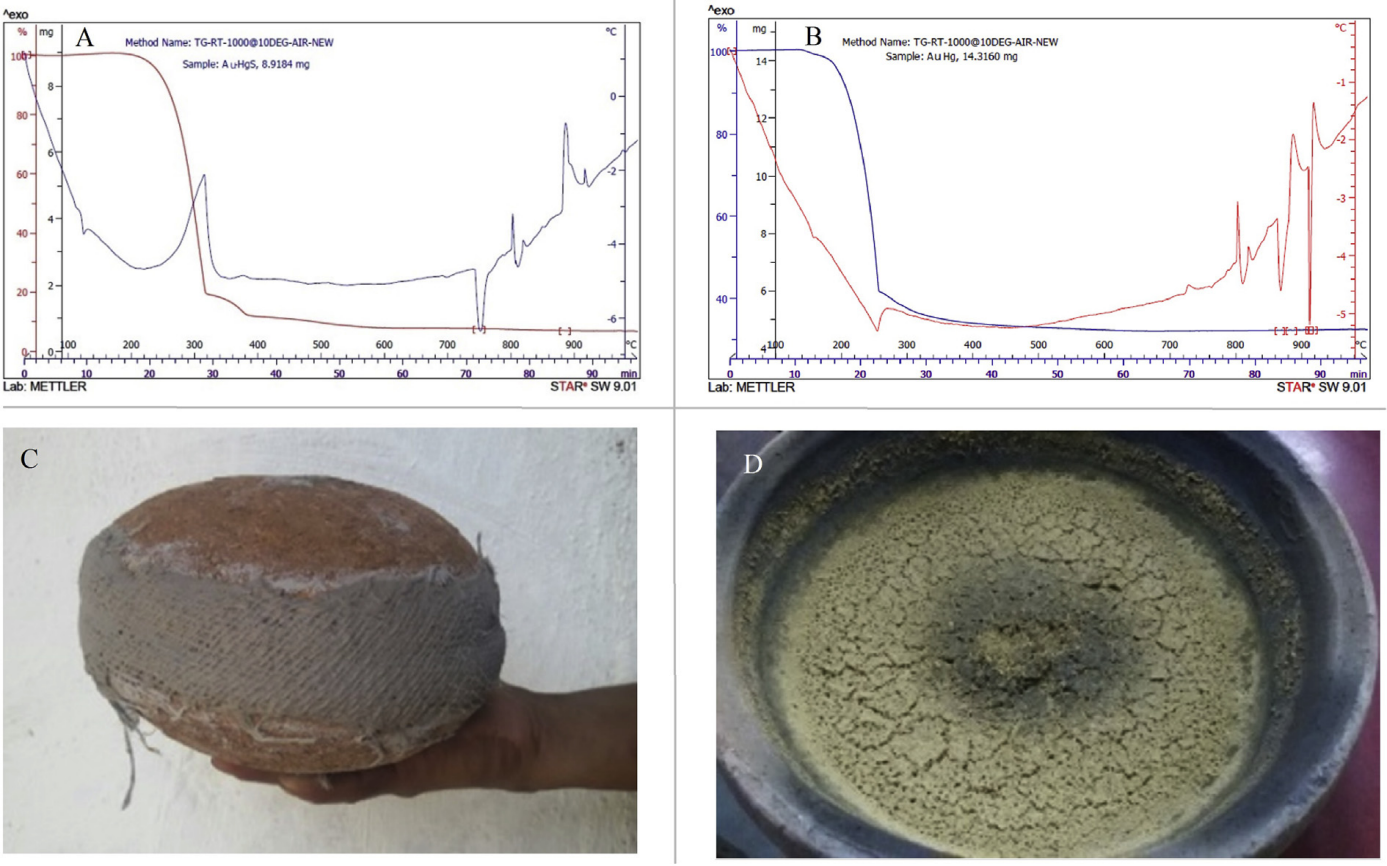
9A- TGA of Au–Hg–S-Citrus juice amalgam in 1:2:16 proportions, 9B- TGA of Au–Hg-Citrus juice in 1:2 proportions, 9C- Closed assembly (*sharav samputa*) in which actual heating takes place in limited air while *bhasma* is prepared, 9D- After 14 heating cycles in *puta*, gold converted to *bhasma*.

Retrospectively, to understand the events in the closed earthen container (*sharav samputa)* during incineration, thermogravimetric analysis (TGA) and differential thermal analysis (DTA) of Au–Hg-Citrus amalgam (1:2) and Au–Hg–S-Citrus (1:2:16) compound (made by trituration) were carried out separately (Fig. 9A and B). Au–Hg amalgam was tested for comparison with actual Au–Hg–S combination, for comparison by the thermo-analytic technique. Of course, the actual setting of incineration using traditional *puta* was not duplicated here [Fig. 9C and D]; however, the thermo-analytical technique was used to assess major endothermic and exothermic reactions of elements in the amalgamate, to get a rough idea of the phenomenon. From the DTA, it is evident that at around 750 °C, the gold in the amalgamation shows endothermic reaction and possibly at 750 °C to 900 °C, gets converted to light brown colored powder i.e., *bhasma*. Due to compounding of Au–Hg-Citrus probably before the melting point, metallic gold is converted in *bhasma* form. Just to check the difference, DTA of Au–Hg-Citrus amalgam was compared which shows a different behavior, showing low (inverted) peak at 850 °C, unlike the Au–Hg–S compound. Citric acid from juice of *C.* *medica* acts as reducing agent for decomposition of amalgam of Au–Hg into Au particles.

The maximum temperature of traditional kiln (*puta*) for making *swarnabhasma* is supposed to reach till 800 to 900 °C. The melting point of gold is 1064 °C when it is in metal state. It appears that the whole of Hg and S transform from the solid state to gaseous state before 300 °C. As per the experience of Ayurvedic physicians, if calcination is carried out at a higher temperature (up to 1500 °C), there cannot be any formation of particles of *swarnabhasma*, and gold reverts in metallic state after calcination. Being a noble metal, gold does not succumb to oxidation or sulfide formation. The peak at 750 °C indicates major endothermic peak, where conversion may be taking place into powder form (*bhasma*) from gold. This endothermic peak is absent in Fig. 9B. The major exothermic peak at 300 °C in Fig. 9A indicates boiling of Hg. Most of the conversion of gold into *bhasma* might be taking place around 750–900 °C. The trituration process in iron mortar pestle after incineration facilitates breakdown of particles. The amalgamation is decomposed at 300 °C and further at 400 °C where exothermic peak due to oxidation of S and evaporation of Hg.

It is presumed that at around 300 °C, all the mercury might leave the amalgamate, escaping out of the *bhasma*. Due the *rasashastriya* purification i.e., *shodhan* process there might be alteration in melting point and boiling point of mercury; however, this can be confirmed by further systematic research to understand effects of *shodhan*.

With this study, the nature of particles of *swarnabhasma* and their size is evident. The particles are in 5–20 nm range which is smaller than that of human cells. In a previous pilot study, bioavailability of gold *bhasma* has been assessed in humans [31]. Particle size is a crucial factor that regulates circulation and navigation of nano-materials in blood stream, penetration across physiological drug barriers, site and cell-specific localization and induction of cellular responses [32].

Apart from gold, the *swarnabhasma* marked presence of other elements in smaller amounts. This included Ag, Al, Ca, Cu, Fe, K, Mg, Mn, Na, P, Si, Sr, Ti, and Zn in traces. Organic and inorganic drugs used during the processing of raw materials (*A. sativum*, rock salt, *B. variegata*, cow’s milk, cow’s ghee) and lemon juice used during amalgamation of Au–Hg, contributed to the presence of these elements in the final *bhasma* of gold. Apart from gold, silicon showed its presence in maximum amount. Silicon is the second-most abundant element in the earth’s crust [33] marking its presence in soil and plants. Iron mortar pestle (*khal*) is used for the preparation of amalgam and further pounding of calcined part after each calcination, which may have contributed to traces of iron into the formulation.

*Swarnabhasma* is a *rasashastriya* medicine which is in use since ancient times for medicinal purposes. *Swarnabhasma* is reported to promote longevity, combat aging process, enhance strength and potency. It has been used as a tonic, hepatotonic, cardiostimulant, nervine tonic, detoxifier, and an anti-infective drug [1]. It is also used for treating anemia, dyspepsia, epilepsy, neurasthenia, memory loss, bronchitis, asthma, tuberculosis, and rheumatic arthritis [1]. This indicates the diverse target action of the drug. The exact mechanism of action of this drug is yet to be explored. Gold *bhasma* has been investigated in a preliminary study for cancer management [34]. Various studies conducted globally, have focused on the use of gold nanoparticles in the management of cancer [[35], [36], [37], [38], [39], [40]]. Its bioavailability, cellular entry *in vivo,* and response of tissues to it needs exploration. The claimed action of gold against toxins by *Charak* *Samhita*, needs to be explored through research. The challenge of toxicity issues of contemporary AuNPs encourage scientists for exploration of new methods for synthesis of AuNPs [41]. On the contrary Ayurvedic *bhasma,* when prepared correctly as per guidelines in *rasashastra* are safe and time-tested. Some novel approaches using gold *bhasma* have been investigated in preclinical and pilot human trials on breast cancer therapy [42].

Due to the rapid development in technologies for the chemical synthesis of gold nanoparticles over recent years, a great variety of particles with different sizes, shapes, structures, and optical properties are now available to contemporary researchers. As safety is the prime concern of nanotechnologists, Ayurvedic formulations must be explored scientifically for in-depth knowledge and more judicious use. Exploration of various traditional methods of preparation of calcined gold can help to enhance our knowledge about gold’s immense pharmacological actions and its therapeutics.

## 5 Conclusion

FE-TEM and XRD confirmed that the investigated *swarnabhasma* comprises of pure gold nanoparticles. FE-TEM showed presence of spherical polycrystalline nanoparticles of pure gold, having 5–20 nm diameter in agglomerated morphology. It is concluded that mercury assists the conversion of gold into *bhasma*, and does not appear in *swarnabhasma*, as proved by EDXRF and ICP-AES. This highlights the important catalytic role of mercury. Due to the presence of gold nanoparticles, further scientific exploration of *swarnabhasma* is necessary for the potential applications in cancer management and other ailments. It must be mentioned here that the results obtained are specific to the investigated sample prepared with specific ingredients and process. Results cannot be generalized for all samples of *bhasma* of gold.

## Source(s) of funding

Bharati Vidyapeeth (Deemed to be University), Pune, (Grant 929).

## Conflict of interest

The authors SN, RG and KT are paid employees of and hence are connected with Shree Dhootapapeshwar Limited, the industry which provided the drug in this study.

## Author contributions

**Trupti Patil-Bhole**: conceptualization, methodology, investigation, data curation, original draft preparation, **Asmita Wele**: methodology, supervision, **Ramacharya Gudi**: resources, **Kapil Thakur**: resources, **Shailesh Nadkarni**: resources, **Rajendra Panmand**: investigation, formal analysis, **Bharat Kale**: methodology, investigation, formal analysis.
